# Dose-dependent effects, safety and tolerability of fenugreek in diet-induced metabolic disorders in rats

**DOI:** 10.1186/1476-511X-10-240

**Published:** 2011-12-21

**Authors:** Etsuko Muraki, Yukie Hayashi, Hiroshige Chiba, Nobuyo Tsunoda, Keizo Kasono

**Affiliations:** 1 Department of Clinical Dietetics & Human Nutrition, Faculty of Pharmaceutical Sciences, Josai University, Saitama 350-0295, Japan; 2 Koken Medicare, Saitama 336-0018, Japan

**Keywords:** fenugreek, high-fat high-sucrose diet, diet-induced metabolic disorders, rats

## Abstract

**Background:**

We previously reported that fenugreek (*Trigonella foenum-graecum *L.) improved diet-induced metabolic disorders in rats. The purpose of the present study was to examine the dose-dependent effects, safety and tolerability of fenugreek.

**Methods:**

The diets used in this study were the high-fat high-sucrose diet (HFS; lard 50%kcal, sucrose 25%kcal) as a control (Ctrl group) or the HFS containing 0.25% (VL group), 1.25% (L group), 2.50% (M group), 5.00% (H group) or 12.30% (VH group) fenugreek based on the modified version of the AIN-93G purified diet.

**Results:**

Fenugreek dose-dependently reduced the hepatic triglyceride and total cholesterol levels. Fenugreek also dose-dependently increased the excretion of cholesterol and total bile acids into the feces. However, the glucose tolerance showed no significant change by fenugreek administration. The VL and L groups did not significantly change triglyceride or total cholesterol levels in the liver. The VL group showed no increase in excretion of triglyceride, total cholesterol or bile acids in the feces. The VH group showed appetite reduction and diarrhea, while no adverse effect or symptoms were observed in the M group.

**Conclusion:**

These results suggest that fenugreek inhibited lipid accumulation in the liver by increasing the lipid excretion in the feces. The effective, safe and tolerable dose of fenugreek was found to be around 2.50% (w/w).

## Background

Recently, the incidence of lifestyle-related diseases including metabolic disorders has been expanding in parallel with rapid economic development in advanced countries. The principle therapeutic policy for these diseases lies in the improvement of dietary habits and the increase of physical activity. However, it is difficult to control and modify one's usual life style. In that case, functional foods with preventive and therapeutic effects on metabolic disorders are very helpful for the improvement of lifestyle-related diseases. On the other hand, the excessive intake of some functional foods may result in adverse effects. However, a lot of such functional foods are actually available at the market, though their safety has not been confirmed [1].

It has been reported that the active components of fenugreek, 4-hydroxyisoleucine and galactomannan, lowered blood glucose levels and improved lipid metabolism *in vivo *[2-5]. Fenugreek also contains dioscin, a steroidal saponin, and trigonelline, an alkaloid. These phytoestrogens have estrogen-like activities *in vitro *[6,7]. In addition, fenugreek contains a large quantity of insoluble dietary fibers. These active components might cause several adverse reactions including hypoglycemia, diarrhea and galactorrhea, when an excessive amount of fenugreek was ingested [2,8,9].

The purpose of this study was to examine the dose-dependent effects, safety and tolerability of fenugreek.

## Methods

### Animals and housing

Three-week-old male, Sprague-Dawley rats were obtained from CLEA (Tokyo, Japan). Rats were housed individually in stainless steel wire-bottom cages in a room maintained at 22 ± 2 °C and 55 ± 5% relative humidity with a 12 h cycle of light and dark. Rats were given free access to tap water throughout the experiment. They were fed a commercial diet (CE-2; CLEA) for 1 wk prior to the experiment. The experimental protocol was approved by the Institutional Animal Care and Use Committee of Josai University.

### Experimental design

The rats were randomly divided into six groups of 6 rats each. Each group was fed either the high-fat high-sucrose diet (HFS; lard 50%kcal, sucrose 25%kcal) based on the modified version of the AIN-93G [10] as a control (Ctrl group) or the HFS containing 0.25% (VL group), 1.25% (L group), 2.50% (M group), 5.00% (H group) or 12.30% (VH group) fenugreek seeds powder (Gaban Co., Tokyo, Japan) for 12 wks (Table 1). Almost the same energy intake was maintained for all groups by adjusting the dietary intake, and the food consumption in all groups except for the VH group was approximately equivalent to the *ad libitum *consumption under these conditions. After 12 wks, the rats were individually transferred into the metabolic cage, and urine and feces were separately collected for 1 day. At the end of the experiment, the rats were killed with pentobarbital sodium (100 mg/kg, ip). Blood samples were collected from the abdominal aorta, and the plasma was separated after centrifugation (2,000 × *g *for 20 min at 4°C) and stored at -30°C until analyzed. After the collection of the blood sample, the liver, interscapular brown adipose tissue (IBAT) and epididymal white adipose tissue (EWAT) were immediately excised, weighed and stored at -30°C for further analyses.

**Table 1:** Experimental diets^1^

(g)	Ctrl	VL	L	M	H	VH
Lard^2^	260.450	260.300	258.100	255.780	251.000	237.700
Milk casein^2^	267.100	266.380	261.900	256.700	246.380	216.500
Sucrose^2^	316.700	316.700	314.700	312.900	309.100	298.600
β-Corn starch^2^	57.698	57.170	55.136	52.343	47.019	31.577
Vitamin mixture^3^	10.000	10.000	10.000	10.000	10.000	9.838
Mineral mixture^4^	35.000	34.923	34.613	34.225	33.450	30.621
Cellulose powder^2^	50.000	48.975	50.000	50.000	50.000	49.190
L-Cystin^5^	3.000	3.000	3.000	3.000	3.000	2.951
t-Butylhydroquinone^5^	0.052	0.052	0.052	0.052	0.051	0.050
Fenugreek^6^	-	2.500	12.500	25.000	50.000	122.974

Ctrl: Control group, VL: Very low dose of fenugreek group (0.25%), L: Low dose of fenugreek group (1.25%), M: Middle dose of fenugreek group (2.5%), H: High dose of fenugreek group (5.0%), VH: Very high dose of fenugreek group (12.3%). ^1^Based on the AIN-93G and modified. ^2,3,4^Purchased from Oriental Yeast Co., Tokyo, Japan. ^3^Vitamin mixture was based on the AIN-93 formation, and added with tartaric acid choline. ^4^Mineral mixture was based on the AIN-93G formation. ^5^Purchased from Wako Pure Chemical Industries, Osaka, Japan. ^6^Purchased from Gaban Co., Tokyo, Japan.

### Oral glucose tolerance tests and intraperitoneal insulin tolerance tests

At the 10th wk, the rats were deprived of food for 12 h. Then they were given oral glucose (2 g/kg body weight) for the oral glucose tolerance test (OGTT) and an intraperitoneal injection of human regular insulin (0.75 U/kg body weight) for the intraperitoneal insulin tolerance test (IPITT). Blood glucose concentrations were measured by Ascensia™ DexterZII (Bayer Medical, Tokyo, Japan) using tail blood samples at 0, 30, 60 and 120 min after glucose administration and at 0, 15, 30, 45, 60 and 120 min after insulin administration. Plasma insulin concentrations were measured using a commercial kit (Rat Insulin ELISA kit, Shibayagi Co., Gunma, Japan) using separated tail plasma samples at 0, 30, 60 and 120 min after glucose administration.

### Blood analysis

Plasma triglyceride, total cholesterol, AST and ALT were measured by colorimetric slides using the Fuji Dri-Chem 3500 (Fujifilm Corp., Tokyo, Japan).

### Hepatic and fecal lipids concentrations

The hepatic and fecal lipids were extracted in accordance with the method of Bligh and Dyer [11] and the method of Folch [12], respectively. Each extract was solubilized by Triton-X100 (Wako Pure Chemical Industries, Osaka, Japan), and the lipids concentrations were determined enzymatically by means of commercial kits (Triglycerides E-Test Wako, Cholesterol E-Test Wako, Total Bile Acid Test Wako; Wako Pure Chemical Industries).

### Statistical analysis

All data are expressed as the mean ± SEM. Statistical analyses were carried out using Statistical Package for Social Sciences (SPSS12.0J for Windows; SPSS Japan, Tokyo, Japan). In the three-group comparison, the effects of treatment were analyzed using one-way analysis of variance (ANOVA), and the differences among means were tested by means of the Tukey's honestly significant difference (HSD) test. In the two-group comparison, the effects of treatment were analyzed through a T-test. In OGTT and IPITT, the effects of treatment were analyzed by means of repeated ANOVA. Differences were considered significant at *P *< 0.05.

## Results

### Effect of fenugreek on the body weight, energy intake, tissue weights and plasma parameters

The body weight gain, the EWAT weight and the IBAT weight decreased significantly in the VH group compared to the other groups. No significant difference was found in the liver weight among the groups (Table 2).

**Table 2:** Effect of fenugreek on the body weight, energy intake, tissue weights and plasma

(g)	Ctrl	VL	L	M	H	VH
Body weight gain (g)	500.6 ± 18.5^a^	491.2 ± 10.2^a^	486.7 ± 18.5^a^	463.9 ± 10.2^a^	480.6 ± 18.5^a^	408.4 ± 10.2^b^
EER^1 ^(mg/kJ)	15.6 ± 0.3^a^	15.3 ± 0.1^a^	15.6 ± 0.3^a^	14.6 ± 0.2^ab^	15.4 ± 0.3^a^	13.4 ± 0.5^b^
Liver weight (g)	19.3 ± 1.1	20.0 ± 0.6	18.3 ± 0.7	17.2 ± 0.6	18.0 ± 0.8	17.3 ± 1.0
EWAT^2 ^weight (g)	15.3 ± 0.5^a^	15.9 ± 0.9^a^	13.6 ± 0.7^ab^	14.5 ± 0.7^a^	16.3 ± 1.4^a^	10.1 ± 1.0b
IBAT^3 ^weight (g)	0.77 ± 0.04	0.76 ± 0.04	0.74 ± 0.05	0.83 ± 0.05	0.78 ± 0.08	0.55 ± 0.03*
Plasma TG^4 ^(mmol/L)	1.64 ± 0.51	1.98 ± 0.61	2.06 ± 0.29	1.79 ± 0.29	3.63 ± 0.73	2.47 ± 0.46*
Plasma TC^5 ^(mmol/L)	1.50 ± 0.14	1.94 ± 0.14	1.73 ± 0.17	1.83 ± 0.17*	1.72 ± 0.13	1.70 ± 0.12
AST (U/L)	66.2 ± 8.0	76.2 ± 10.6	94.3 ± 21.7	98.5 ± 25.7	67.7 ± 9.1	74.8 ± 8.2
ALT (U/L)	17.3 ± 0.7	21.2 ± 3.2	22.5 ± 3.9	25.0 ± 4.6	19.2 ± 2.4	22.2 ± 1.4
AST/ALT^6^	1.08 ± 0.11	1.03 ± 0.04	1.14 ± 0.06	1.05 ± 0.08	1.02 ± 0.09	0.95 ± 0.07

Ctrl: Control group, VL: Very low dose of fenugreek group (0.25%), L: Low dose of fenugreek group (1.25%), M: Middle dose of fenugreek group (2.5%), H: High dose of fenugreek group (5.0%), VH: Very high dose of fenugreek group (12.3%). ^1^EER (mg/kJ) = Body weight gain (mg)/Total food intake (kJ). ^2^EWAT: Epididymal white adipose tissue. ^3^IBAT: Interscapular brown adipose tissue. ^4^TG: Triglyceride. ^5^TC: Total cholesterol. ^6^The relative values for Normal rats' data; AST/ALT = AST (U/L)/ALT (U/L)/Normal rats' data. Values are expressed as means ± SEM for 6 rats. Means without a common superscript letter are significantly different, *P *< 0.05 (ANOVA). Asterisks indicate a difference from Ctrl: *P *< 0.05 (t-test).

No significant difference was found in AST, ALT levels or AST/ALT in the liver among the groups (Table 2). Thus fenugreek intake did not show any liver injury.

### Effects of fenugreek on glucose tolerance

The fasting blood glucose levels decreased significantly in the M and VH groups compared to the Ctrl group, whereas no significant difference was found in the fasting plasma insulin levels or insulin resistance index, HOMA-IR (homeostasis model assessment as an index of insulin resistance), among groups (Table 3).

**Table 3:** Insulin resistance index

(g)	Ctrl	VL	L	M	H	VH
Fasting blood glucose (mmol/L)	6.27 ± 0.24^a^	6.19 ± 0.22^ac^	5.50 ± 0.28^ab^	5.20 ± 0.15b^c^	5.60 ± 0.34^ab^	5.03 ± 0.11^b^
Fasting blood insulin (pmol/L)	129 ± 37	167 ± 35	56 ± 27	117 ± 29	94 ± 33	54 ± 10
HOMA-IR^1^	1.00 ± 0.28	1.69 ± 0.49	0.85 ± 0.52	0.78 ± 0.20	0.98 ± 0.36	0.35 ± 0.06

Ctrl: Control group, VL: Very low dose of fenugreek group (0.25%), L: Low dose of fenugreek group (1.25%), M: Middle dose of fenugreek group (2.5%), H: High dose of fenugreek group (5.0%), VH: Very high dose of fenugreek group (12.3%). ^1^Relative values of (fasting blood glucose × fasting blood insulin) compared to the mean value of the Ctrl group. Values are expressed as means ± SEM for 6 rats. Means without a common superscript letter are significantly different, P < 0.05 (ANOVA).

**Figure 1:**
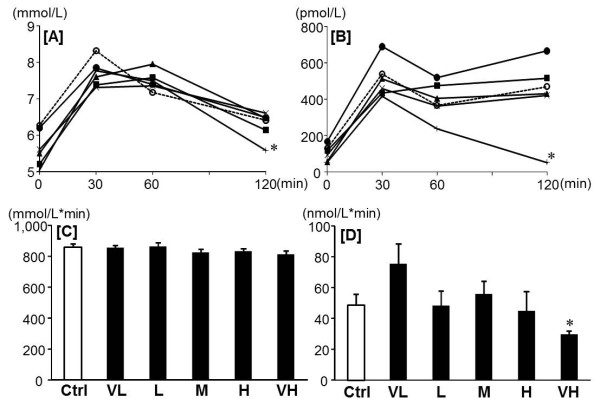
**Effect of fenugreek on the plasma insulin and blood glucose levels in OGTT**. Changes of blood glucose [A], changes of plasma insulin [B], AUC of blood glucose [C], AUC of plasma insulin [D] after oral glucose load (2 g/kg). Ctrl: Control group [○], VL: Very low dose of fenugreek group (0.25%) [●], L: Low dose of fenugreek group (1.25%)[▲], M: Middle dose of fenugreek group (2.5%) [■], H: High dose of fenugreek group (5.0%) [×], VH: Very high dose of fenugreek group (12.3%)[+]. Values are expressed as means for 6 rats [A,B]. Values are expressed as means ± SEM for 6 rats [C,D]. Asterisks indicate a difference from Ctrl: *P *< 0.05 (t-test).

In OGTT, the blood glucose levels at 120 min in the VH group were significantly lower than those in the Ctrl group (Figure 1A), and the plasma insulin levels at 120 min in the VH group were significantly lower than those in other groups (Figure 1B). There was no significant difference of the area under the curve (AUC) of glucose among the groups (Figure 1C), but the VH group showed significant lower AUC of insulin compared to the Ctrl group (Figure 1D).

**Figure 2:**
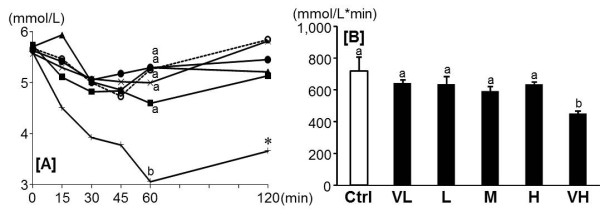
**Effect of fenugreek on blood glucose levels in IPITT**. Changes of blood glucose [A] and AUC [B] after intraperitoneal insulin load (0.75 U/kg). Ctrl: Control group [○], VL: Very low dose of fenugreek group (0.25%) [●], L: Low dose of fenugreek group (1.25%)[▲], M: Middle dose of fenugreek group (2.5%) [■], H: High dose of fenugreek group (5.0%) [×], VH: Very high dose fenugreek group (12.3%)[+]. Values are expressed as means for 6 rats [A]. Values are expressed as means ± SEM for 6 rats [B]. Means without a common superscript letter are significantly different, *P *< 0.05 (ANOVA). Asterisks indicate a difference from Ctrl: *P *< 0.05 (t-test).

In IPITT, the blood glucose levels at 60 and 120 min in the VH group were significantly lower than those of the other groups (Figure 2A). AUC of the blood glucose in the VH group was also significantly lower than those of the other groups (Figure 2B).

### Lipids and bile acid levels in plasma, liver and feces

**Figure 3:**
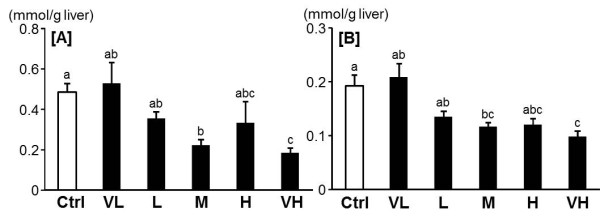
**Effect of fenugreek on the hepatic triglyceride [A] and total cholesterol [B] levels**. Ctrl: Control group, VL: Very low dose of fenugreek group (0.25%), L: Low dose of fenugreek group (1.25%), M: Middle dose of fenugreek group (2.5%), H: High dose of fenugreek group (5.0%), VH: Very high dose of fenugreek group (12.3%). Values are expressed as means ± SEM for 6 rats. Means without a common superscript letter are significantly different, *P *< 0.05 (ANOVA).

**Figure 4:**
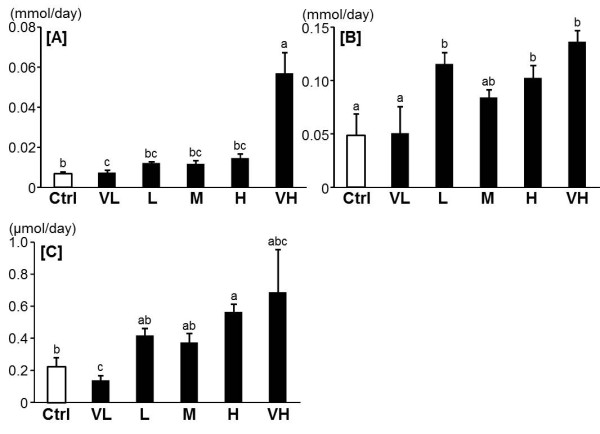
**Effect of fenugreek on the fecal triglyceride [A], total cholesterol [B] and total bile acid secretion [C] levels**. Ctrl: Control group, VL: Very low dose of fenugreek group (0.25%), L: Low dose of fenugreek group (1.25%), M: Middle dose of fenugreek group (2.5%), H: High dose of fenugreek group (5.0%), VH: Very high dose of fenugreek group (12.3%). Means without a common superscript letter are significantly different, *P *< 0.05 (ANOVA).

Although the plasma triglyceride and total cholesterol levels increased slightly in some groups, no dose dependent effect was found (Table 2). However, the hepatic triglyceride and total cholesterol levels decreased significantly in the M and VH groups compared to the Ctrl group (Figure 3). In feces, the triglyceride levels in the VH group increased remarkably compared to the levels the in the other groups. The total cholesterol levels increased significantly in the L group, the H group and the VH group compared to the Ctrl group. Fenugreek elevated the total bile acid levels in feces in a dose-dependent manner (Figure 4).

## Discussion

Hepatic steatosis is a risk factor for liver cirrhosis and atherosclerosis. Both the liver and adipocytes play a major role in the regulation of cellular and circulating serum lipids, predominantly triglyceride and cholesterol. In our previous studies, fenugreek decreased the hepatic triglyceride and total cholesterol levels in normal rats fed with a high-fat high-sucrose diet [13,14]. The present study investigated dose-dependent effects of fenugreek and its safety and tolerability.

The body weight gain and EWAT and IBAT weights in the VH group decreased significantly as compared with all of the other groups. In a pair feeding procedure, the VH group showed slightly less food intake than the other groups. It has been reported that the reduced food intake in the presence of soluble fibers such as galactomannan contained in fenugreek was caused by delaying gastric emptying and promoting satiety. In our study, the ratio of body weight gain to food intake was similar among the groups [5]. However, the VH group showed significant lower levels of the energy efficiency ratio (EER) compared to the Ctrl group. Therefore, we suspect that the decreases in body and tissue weight were caused by the decrease of EER rather than the decrease in energy intake.

Fenugreek dose-dependently reduced the hepatic triglyceride and total cholesterol levels. Whereas the fecal triglyceride excretion levels rose significantly only in the VH group, fenugreek dose-dependently augmented the fecal total cholesterol and the bile acid excretion levels. The plasma triglyceride and total cholesterol levels were not significantly different among the groups. These results suggest that the mechanism underlying the inhibition of lipid accumulation in the liver and the adipose tissue would have enhanced the total cholesterol and the bile acid excretion in feces. Likewise, the increase of triglyceride excretion led to these results in the VH group.

Saponins, such as diosgenin contained in fenugreek, form large micells from bile acid and saponin molecules in the small intestine, and these micelles inhibit the cholesterol absorption by directly excreting cholesterol in feces [15,16]. Diosgenin also reduces the triglyceride content and mRNA expression levels of lipogenic genes (FAS, SCD-1 and ACC) and suppresses LXRα transactivation. This leads to down-regulation of both the mRNA and protein expression levels of SREBP-1c in HepG2 cells [17]. These results obtained from the previous reports supported that fenugreek attenuated lipid accumulation in the liver by down-regulating lipid synthesis as well as increasing lipid excretion in the feces.

The fasting blood glucose levels decreased significantly in the M and VH groups as compared with the Ctrl group, but the fasting plasma insulin levels did not differ significantly among the fenugreek-administered groups as compared with the Ctrl group. HOMA-IR was not significantly different among the groups. Therefore, in these experiments, fenugreek did not affect the glucose tolerance. However, some improving effects on the glucose metabolism and glucose tolerance of fenugreek have been reported. For example, 4-hydroxyisoleucin contained in fenugreek stimulates insulin secretion [2,18]. In OGTT in our experiment, the fenugreek administration did not significantly change the plasma insulin levels or blood glucose levels except at 120 min in the VH group. Further, fenugreek inhibited insulin secretion only in the VH group. It has been reported that fenugreek also enhances insulin sensitivity through the activation of insulin signaling at an early stage in peripheral tissues and liver [19]. This effect is brought by the activation of glucose and lipid metabolism with up-regulation of several enzymes [19-23] and the increase of glycogen synthesis in the muscle and liver [22-24]. In addition, diosgenin enhances the peroxisome proliferator-activated receptor-γ (PPARγ) level in EWAT and promotes both the adipocyte differentiation and the size reduction. As a result, the secretion of monocyte chemoattractant protein-1 (MCP-1) in adipocytes is suppressed, while the secretion of adiponectin is promoted, and the inflammation in adipose tissue is inhibited [18,25]. Therefore, we assume that fenugreek activated firstly insulin sensitivity rather than insulin secretion in the relatively mild metabolic disorders we generated in rats through high-fat high-sucrose diets. The VH group showed appetite reduction and diarrhea, while no rats in the M group showed any adverse effects or symptoms.

These results suggest that fenugreek dose-dependently inhibited lipid accumulation in the liver by increasing the lipid and bile acids excretion in the feces, and that an effective, safe and tolerable dose of fenugreek was around 2.50% (w/w).

## Competing interests

The authors declare that they have no competing interests.

## Authors' contributions

EM conceived the study and its design, wrote the manuscript, and carried out the experiments. YH carried out experiments and analyzed data. HC and NT participated in study design and helped to draft the manuscript. KK conceived the study, participated in its design and helped to draft the manuscript. All authors read and approved the final manuscript.

## Authors' information

Department of Clinical Dietetics & Human Nutrition, Faculty of Pharmaceutical Sciences, Josai University, Saitama, Japan.
